# Evaluation of Apparent Metabolizable Energy and Apparent Ileal Amino Acid Digestibility of Spirulina (*Arthrospira platensis*) in Broiler Chickens and Laying Hens

**DOI:** 10.3390/ani14223343

**Published:** 2024-11-20

**Authors:** Taylor K. O’Lear Reid, Katherine E. Gardner, Kayla L. Paglia, Alexandra C. M. Ulans, Ruth E. Spierling, Mark S. Edwards, Tryg J. Lundquist, Zach D. McFarlane, Siroj Pokharel, Darin C. Bennett

**Affiliations:** 1 Animal Science Department, California Polytechnic State University, San Luis Obispo, CA 93407, USA; tkolearr@ncsu.edu (T.K.O.R.); katherinegardner813@gmail.com (K.E.G.); kaylaerath@yahoo.com (K.L.P.); alexandraulans@vt.edu (A.C.M.U.); msedward@calpoly.edu (M.S.E.); zmcfarla@calpoly.edu (Z.D.M.); spokhare@calpoly.edu (S.P.); 2 Prestage Department of Poultry Science, North Carolina State University, Raleigh, NC 27695, USA; 3 Department of Animal and Poultry Sciences, Virginia Tech, Blacksburg, VA 24061, USA; 4 MicroBio Engineering Inc., San Luis Obispo, CA 93406, USA; ruthspierling@lacsd.org (R.E.S.); tlundqui@calpoly.edu (T.J.L.); 5 Los Angeles County Sanitation Districts, Whittier, CA 90745, USA; 6 Civil and Environmental Engineering Department, California Polytechnic State University, San Luis Obispo, CA 93407, USA

**Keywords:** spirulina meal, apparent metabolizable energy, apparent ileal amino acid digestibility, broiler chicken, laying hen

## Abstract

Our study objective was to determine the apparent metabolizable energy (AME) and apparent ileal amino acid digestibility (AIAAD) of spirulina fed to broiler chickens and laying hens using the difference method. In both experiments, birds were either fed corn-soybean meal basal diets, containing no spirulina and formulated to provide the nutrient requirements of either broilers or layers, or fed test diets consisting of 25% spirulina and 75% of the appropriate basal diet. Titanium dioxide was added to all diets as an indigestible marker. The diets were fed to 10 replicate cages/treatment of broilers and 15 replicate cages/treatment of laying hens. The AME of spirulina for broilers was significantly lower (*p* < 0.05) (2368 ± 104 kcal/kg, as fed) than for laying hens (3144 ± 173 kcal/kg, as fed), suggesting bird type and age may influence energy utilization. The AIAAD of spirulina fed to broiler chickens did not differ from that of laying hens (*p* > 0.05), except for valine, alanine, and glycine, which were all significantly higher in laying hens (*p* < 0.05). Overall, the AIAAD for nonessential amino acids averaged 81.1%, with no significant difference between essential and nonessential amino acids. Differences in spirulina nutrient content cited in the literature support further research to determine the optimal inclusion of this alternative ingredient in broiler and layer diets.

## Simple Summary

A growing world population has increased the demand for animal protein, and the poultry industry is a major contributor to meeting this demand. Increasing feed costs and unreliable availability have prompted the poultry industry to identify and evaluate alternative feed ingredients. In this study, we evaluate the energy and amino acid availability of spirulina (*Arthrospira platensis*), a filamentous blue-green microalgae. The results show that spirulina is a high-quality feed ingredient for possible inclusion in poultry diets.

## 1. Introduction

The growing world population has increased the demand for animal protein. The poultry industry is a major contributor to meeting this demand by providing high-quality protein (meat and eggs) with an efficient feed-to-product conversion ratio. However, feed cost is the largest single item in poultry production, typically accounting for 60–70% of the total production cost [1]. The poultry industry relies on a few major ingredients for feed formulation. Corn and other cereal grains, such as wheat and barley, are the main energy sources used, while soybean meal and canola meal are the major protein sources utilized [1]. However, increasing costs and competition with other sectors [2] have prompted the poultry industry to identify and evaluate alternative feed ingredients that meet the energy and nutritional requirements of poultry. In addition, the poultry industry is always seeking new feed ingredients or additives to improve profitability and product quality, such as by improving animal health, productivity, or adding value to the product. Many studies suggest that the addition of various microalgae species to diets can help achieve these goals [3,4,5,6,7].

One promising type of microalgae is spirulina (*Arthrospira* spp.), a filamentous blue-green microalgae that is an excellent source of energy, protein, and essential amino acids for poultry [8,9,10,11,12,13]. Spirulina is also a rich source of carotenoids and n-3 fatty acids [3] and may provide additional value in terms of improving health and immunity [7,14,15,16]. The fast growth rate and relative ease of cultivation has led to large scale production of spirulina in many parts of the world [17,18]. However, the nutrient composition of spirulina can vary [19] depending on the specific species and strain, cultivation method, growing conditions, and other factors [5,17,18]. Therefore, it is important to evaluate the nutritional value and feeding potential of spirulina from various sources for use in broiler and layer production.

Studies have examined the effects of dietary supplementation with spirulina on broiler growth performance and meat quality [6,20,21], and on laying hen performance and egg quality [22,23,24]. An accurate assessment of the energy and amino acid availability of spirulina when fed to poultry is vital to precise diet formulation, which is essential to an evaluation of the potential of spirulina for use in broiler and layer production. However, only a limited number of studies have been conducted on broilers [10,11,12,13], and these values have not been reported for laying hens. The objective of this study was to determine the apparent metabolizable energy (AME) and apparent ileal amino acid digestibility (AIAAD) of spirulina in broiler chicks and laying hens using the difference method [25].

## 2. Materials and Methods

### 2.1. Ethics

This study was conducted at the poultry facilities of the Animal Science Department, California Polytechnic State University. All experimental procedures were reviewed and approved by the California Polytechnic State University Institutional Animal Care and Use Committee (Protocol #1613, 1908).

### 2.2. Spirulina Production

The spirulina (*Arthrospira platensis*) used in this study were locally grown (MicroBio Engineering Inc., San Luis Obispo, CA, USA) in a 200 m^2^ paddle wheel-mixed raceway pond enclosed in a greenhouse. The growth medium was fresh water supplemented with bicarbonate alkalinity and chemical fertilizer for nitrogen and phosphorus. After screen harvesting, the spirulina was rinsed with fresh water, dewatered, and solar-dried. Spirulina was harvested during the day, and drying times were dependent on the ambient temperature and wind conditions, typically taking less than a week. The material was rotated daily to promote even drying and ensure proper air circulation. The dried spirulina was then ground to a particle size of ~1 mm using a Wiley mill.

### 2.3. Experiment 1—Broiler Chick Digestibility Trial

Day-old unsexed Ross 708 broiler chicks were obtained from a commercial hatchery (Foster Farms, Fresno, CA, USA) and reared in a floor pen (0.96 sq. ft./bird). All birds were vaccinated at hatching against Newcastle disease, Marek’s disease, and infectious bronchitis at the hatchery. Throughout the pre-experimental period, birds were offered a corn-soybean meal-based grower diet (Table 1) and water ad libitum.

At 25 days of age, 80 birds were weighed, sorted based on body weight, and placed into 20 wire cages (2′ × 2′, 4 birds/cage, 1.0 sq. ft./bird) with trough feeders and nipple drinkers. Cages of birds were allocated to one of two dietary treatments (10 cages/diet): (1) a control diet (Basal corn-soybean meal-based grower diet; Table 1) or (2) a test diet (75% basal grower diet +25% spirulina). The basal diet and spirulina were combined in a Hobart 60 Qt mixer. Titanium dioxide (TiO_2_; 5 g/kg) was added to both diets as an indigestible marker. Both diets were fed as a mash. The room temperature was maintained at 22 °C, and the lighting was 16 L:8 D.

After a 5-day diet adaptation period, excreta were collected daily from each cage over a 3-day period to evaluate the apparent metabolizable energy (AME). Daily excreta collections were pooled within a replicate cage. Birds were then euthanized by CO_2_ inhalation, and the digesta contents of the lower two-thirds of the ileum were collected and pooled by cage. The ileum was defined as the portion of small intestine extending from Meckel’s diverticulum to a point 40 mm proximal to the ileocecal junction.

### 2.4. Experiment 2—Laying Hen Digestibility Trial

Thirty Lohman LSL-Lite laying hens of 80-weeks of age were randomly selected from the California Polytechnic State University Layer flock. Hens were randomly assigned to individual wire cages (2′ × 2′) with trough feeders and nipple drinkers. Hens received one of two diets (15 cages/diet): (1) a control diet (basal corn-soybean meal-based layer diet) that was fed to the hens during the pre-experimental period; Table 1 or (2) a test diet (75% basal layer diet +25% spirulina). The basal diet and spirulina were combined in a Hobart 60 Qt mixer. Titanium dioxide (TiO_2_, 5 g/kg) was added to both diets as an indigestible marker. Both diets were fed as a mash. The room temperature was maintained at 22 °C, and the lighting was 16 L:8 D.

After a 5-day diet adaptation period, excreta were collected daily from each cage over a 24-h period to evaluate metabolizable energy. Daily excreta collections were pooled within a replicate cage. Birds were euthanized by CO_2_ inhalation, and their ileal contents were collected. The ileum was defined as that portion of small intestine extending from Meckel’s diverticulum to a point 40 mm proximal to the ileo-cecal junction.

### 2.5. Nutrient Analysis

Excreta and ileal samples from both trials were dried at 50 °C and ground to pass through a 0.5-mm sieve. To ensure an adequate amount of dried ileal sample for amino acid and titanium analysis, 3–4 cages per treatment were pooled, thus reducing the sample size for AIAAD analysis. All samples were stored at −20 °C for later nutrient analyses.

Gross energy was measured in the California Polytechnic Animal Science Nutrition Lab using an isoperiobol bomb calorimeter (model 6200, Parr Instrument Company, Moline, IL, USA) standardized with benzoic acid. All nutrient analyses (proximate analysis, amino acid content, and titanium) were conducted by the Agricultural Experiment Station Chemical Laboratories of the University of Missouri–Columbia, according to AOAC [26]. Titanium dioxide was measured and calculated using the methods described by Myers et al. [27]. Spirulina was analyzed for dry matter (method 934.01), crude protein (LECO, method 990.03), crude fat (method 920.39), ash (method 942.05), neutral detergent fiber (method 2002.04), acid detergent fiber (method 973.18), amino acid content (method 982.30), and gross energy. Samples of the diets and ileal digesta were analyzed for dry matter, titanium, and amino acid content. Samples of the diets and excreta were analyzed for dry matter, titanium, and gross energy.

### 2.6. Calculations and Statistical Analyses

The apparent metabolizable energy (AME, kcal/kg) value of the control diet (AMEControl diet) was calculated as:

AME_control diet_ = GE_control diet_ − (GE_excreta_ × ([Ti]_diet_/[Ti]_excreta_)) (1)

where GE is the gross energy of the diet (GE_control diet_) and excreta (GE_excreta_), and [Ti] is the titanium concentration of the diet (([Ti]_diet_) and excreta ([Ti]_excreata_). The AME value of the spirulina (AME_Spirulina_) was then calculated as follows:

AME_Spirulina_ = [AME_Test diet_ − (AME_Control diet_ × 0.75)]/0.25 (2)

where AME_Test diet_ and AME_Control diet_ are the AME of the test diet and control diet, respectively.

The apparent ileal amino acid digestibility (AIAAD) value of the diets (AIAAD_diet_) was based on the use of the indigestible marker and was calculated using the following formula:

AIAAD_diet_ = {1 − [(AA/Ti)_ileal digesta_/(AA/Ti)_diet_]} × 100 (3)

where (AA/Ti)_ileal digesta_ is the ratio of the amino acid and titanium concentrations in the ileal digesta and (AA/Ti)diet is the ratio of the amino acid and titanium concentrations in the diet. The apparent ileal amino acid digestibility value of the spirulina was calculated using the following formula:

AIAAD_Spirulina_ = [AIAAD_Test diet_ − (AIAAD_Control diet_ × 0.75)]/0.25. (4)

All statistical analyses were performed using JMP Pro 17 (SAS Institute Inc., Cary, NC, USA). Data normality was verified using Shapiro–Wilk normality tests. Differences between the AME and AIAAD of spirulina fed to broilers and layers were determined using two-sample *t*-tests. Statistical significance was accepted at *p* < 0.05. The data are reported as means ± SEM and on an as fed basis.

## 3. Results

The energy and nutrient composition of spirulina used in the current broiler chicken and laying hen digestibility trials were similar and are presented in Table 2 and Table 3, respectively. The AME values of spirulina determined in this study were significantly lower for the broiler chicks (2368 ± 104 kcal/kg, As Fed; 52.2 ± 2.3% of GE) than for the laying hens (3144 ± 173 kcal/kg, As Fed; 68.8 ± 3.8% of GE; Table 4). The AIAAD (%) of spirulina did not differ between the broiler chickens and laying hens, except for valine, alanine, and glycine, which were all significantly higher in laying hens (Table 5). The AIAAD of indispensable amino acids (81.1 ± 1.9%) did not differ significantly from that of dispensable amino acids (78.3 ± 3.1%), averaging 79.9 ± 1.7%.

## 4. Discussion

Interest in spirulina as a possible feed ingredient for use in the poultry industry has increased, due to its fast growth, relative ease of cultivation [17,18], and nutrient composition [5,10,11,12,13]. Spirulina can also provide additional value through its functional properties: improved health and immunity [7,15,16], improved production performance, and improved product quality [6,20,21,22,23,24]. However, the energy and amino acid composition and availability of spirulina needs to be determined if this ingredient is to be used in poultry diets. Therefore, the objective of this study was to determine the AME and AIAAD of spirulina for broiler chickens and laying hens.

### 4.1. Energy and Nutrient Composition

Overall, the gross energy and nutrient composition of spirulina used in the current study (Table 2 and Table 3) were within the range reported in previous poultry nutrient digestibility trials (Table A1). Spirulina (dried) has a dry matter content of 88.6 ± 0.9%, as fed. It has a high gross energy (4588 ± 121 kcal/kg, as fed), crude protein (58.6 ± 1.9%, as fed), and ash content (8.1 ± 0.4%, as fed), all with moderate levels of variation (CV < 12%). The lipid (crude fat) and complex carbohydrate (crude fiber, neutral detergent fiber) content are relatively low, but highly variable (CV > 50%).

### 4.2. Apparent Metabolizable Energy

The AME value of spirulina determined in this study for broiler chickens (Table 4) was lower than that determined in previous studies [10,12,13] (Table A2), despite similar gross energy and nutrient composition (Table A1). The broilers used in this study were 30 days old at the start of excreta collection, while those used in the previous studies were 18–19 days old [10,12,13], suggesting a possible age-related effect. Khalil et al. [28] found that the AME of soybean meal and canola meal for broilers decreased quadratically as the birds grew. The AME of spirulina determined for the adult 80-week-old laying hens (Table 4) in the present study was similar to that determined for 1-week old male Leghorn chicks [8]. Both values were greater than that determined for the broilers, suggesting possible differences between ‘Leghorn-type’ chickens and ‘broiler-type’ chickens. Future studies should examine the effects of age and chicken type on the AME of spirulina.

The variation in the AME of spirulina fed to chickens across the various studies was moderate (CV 13%; Table A2). In the absence of a more detailed examination of the effects of age, chicken type, and variation in nutrient composition on the AME of spirulina, we recommend using an average AME value of 2882 ± 153 kcal/kg, as fed, or 62.9 ± 3.3% of the gross energy in diet formulations. This is comparable to the average AME value of soybean meal determined across various studies: 2746 ± 40 kcal/kg, as fed or 63.6 ± 11.2% of gross energy (Bennett, unpublished meta-analysis).

### 4.3. Apparent Ileal Amino Acid Digestibility

The amino acid composition (Table 2 and Table 3) and availability (AIAAD; Table 5) of spirulina determined in this study indicate that spirulina is a high-quality protein source with a well-balanced amino acid profile, supporting the findings of previous studies [12,13]. The AIAAD of spirulina determined for broiler chickens in the present study were comparable to those of Tavernari et al. [12] and Mullenix [13]. There were discrepancies among the three studies (Table A3), possibly reflecting differences in the spirulina used, ages and genetics of the broiler chickens, or in the methods used to determine AIAAD. However, for most of the amino acids the variation among studies was low.

This study was the first to determine the AIAAD of spirulina using laying hens. We found that these values did not differ from those determined for broiler chickens, except for valine, alanine, and glycine, which were all significantly higher in laying hens (Table 5). We also found no overall statistical difference in the AIAAD of indispensable and dispensable amino acids, which averaged 79.9 ± 1.7%.

**Figure 1:**
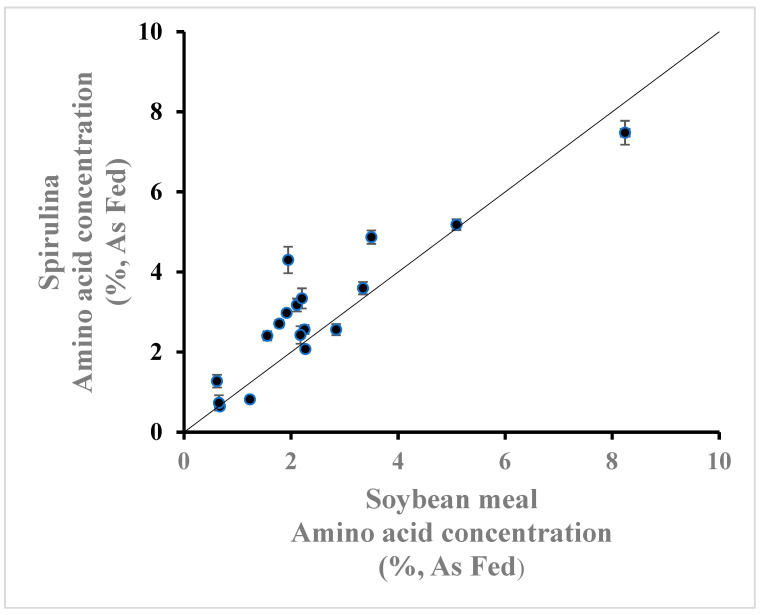
Total amino acid concentration (%, As Fed) of spirulina (Table A1) compared with that of soybean meal (Bennett, unpublished meta-analysis containing 56 values from 31 studies published between 2012 and 2023). Line of equality is provided.

**Figure 2:**
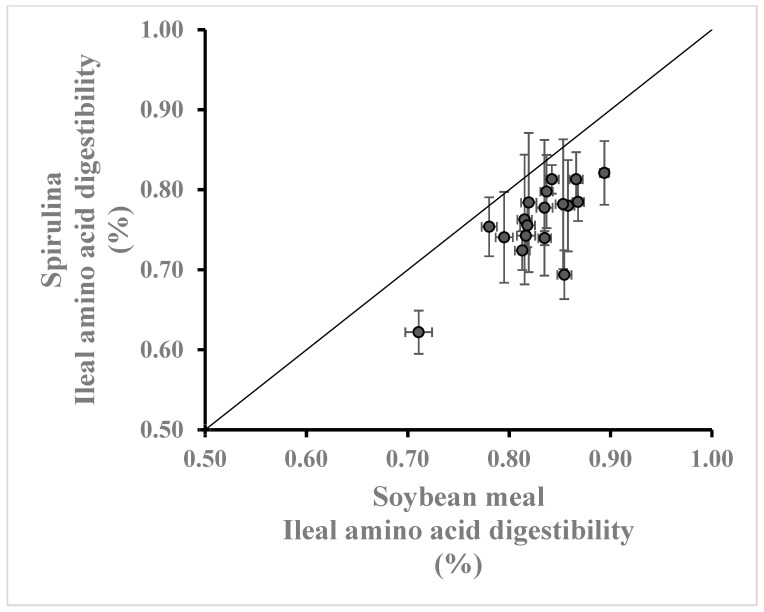
Apparent ileal amino acid digestibility (%) of spirulina (Table A3) compared with that of soybean meal fed to broiler chicks and laying hens (Bennett, unpublished meta-analysis containing 56 values from 31 studies published between 2012 and 2023). Line of equality is provided.

**Figure 3:**
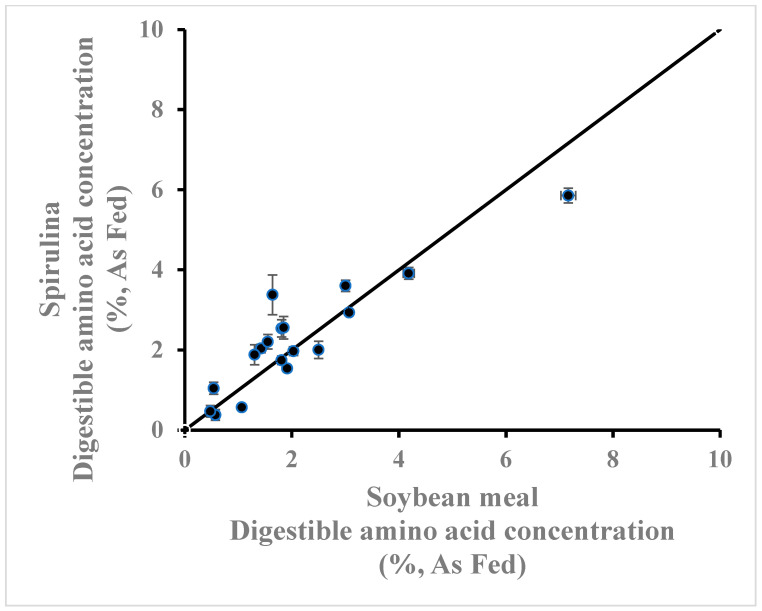
Apparent ileal digestible amino acid concentration (%, As Fed) of spirulina (calculated from Table A1 and Table A3) compared with that of soybean meal (Bennett, unpublished meta-analysis containing 56 values from 31 studies published between 2012 and 2023). Line of equality is provided.

Many studies have suggested that spirulina has the potential to partially replace soybean meal, the major protein source used in poultry diets [29,30]. The results of the current study and those of previous digestibility studies support this conclusion. Comparing the amino acid composition and availability of spirulina (Table A1 and Table A3) to those of soybean meal (Bennett, unpublished meta-analysis containing 56 values from 31 studies published between 2012 and 2023), indicates that the amino acid concentration of spirulina is equal or greater than that of soybean meal (Figure 1), but with a lower IAAD (Figure 2). Consequently, the digestible amino acid concentration of spirulina is equal or greater to that of soybean meal (Figure 3).

## 5. Conclusions

The AME and AIAAD of spirulina when fed to broiler chicks and laying hens were determined. These values are essential to formulate diets containing spirulina that meet the nutrient requirements of poultry. The results show that spirulina has an AME value and digestible amino acid concentration that is equal to or superior to that of soybean meal. These findings show that spirulina is a high-quality feed ingredient for possible inclusion in poultry diets.

## Figures and Tables

**Table 1:** Ingredient composition and calculated analysis of the basal diets used in the broiler chick (experiment 1) and laying hen (experiment 2) digestibility trials. Diets were fed as a mash.

	Boiler Chick Grower Diet	Layer Hen Diet
Ingredients (%, As Fed)		
Corn	53.0	58.3
Soybean meal	34.3	11.7
Corn DDGS	5.0	10.0
Vegetable fat	3.3	
Limestone	1.2	8.1
Mono-Dicalcium phosphate blend	1.4	0.90
Salt	0.32	0.25
DL-Methionine	0.35	0.14
L-Lysine HCl	0.12	-
L-Threonine	0.11	-
Vitamin premix ^1^	0.50	0.38
Trace mineral premix ^2^	0.30	0.11
Nicarb 25%	0.04	-
Calculated analysis (%, As Fed)		
AME (kcal/kg)	3100	2725
Dry matter	88.0	87.9
Crude protein	21.5	14.3
Crude fat	6.2	3.5
NDF	9.0	13.1
Ca	0.87	3.3
Available *p*	0.43	0.35
Methionine	0.67	0.37
Methionine + Cysteine	0.99	0.55
Lysine	1.31	0.69
Threonine	0.89	0.51

^1^ Vitamins supplied per kg premix: vitamin A, 4,000,000 IU; vitamin D_3_, 1,000,000 IU; vitamin E, 4000 IU; vitamin K_3_, 1200 mg; thiamine, 400 mg; riboflavin, 1600 mg; niacin, 12,000 mg; pantothenic acid, 3200 mg; vitamin B6, 800 mg; biotin, 10 mg; folic acid, 200 mg; vitamin B12, 6 mg; Choline chloride, 160 g. ^2^ Trace minerals supplied per kg premix: manganese (MnSO_4_), 40 g; zinc (ZnSO_4_), 24 g; iron (FeSO_4_), 10 g; copper (CuSO_4_), 2 g; selenium (Na_2_SeO_3_), 0.1 g.

**Table 2:** Analyzed energy and nutrient composition of the spirulina and experimental diets (as fed basis) used in the broiler chick digestibility trial. Diets were fed as a mash.

Composition (%, As Fed)	Spirulina	Control Diet ^1^	Test Diet ^2^
Dry matter (%)	86.2	88.2	87.6
Gross energy (kcal/kg)	4539	3930	3123
Apparent metabolizable energy (kcal/kg)	2368	2946	2798
Crude protein (%)	61.0	21.3	31.6
Crude fat (%)	3.2	4.1	3.9
Neutral detergent fiber (%)	3.3	9.6	9.4
Acid detergent fiber (%)	1.3	2.0	5.2
Ash (%)	8.1	6.2	6.9
Non-fiber carbohydrate (%, calculated by difference)	10.6	47.0	35.8
Total Indispensable amino acids			
Arginine (%)	3.34	1.41	1.97
Histidine (%)	0.85	0.59	0.68
Isoleucine (%)	3.45	0.99	1.48
Leucine (%)	5.14	1.88	2.51
Lysine (%)	2.78	1.32	1.73
Methionine (%)	1.46	0.71	1.16
Phenylalanine (%)	2.72	1.10	1.49
Threonine (%)	2.74	0.95	1.24
Tryptophan (%)	0.62	0.26	0.33
Valine (%)	3.75	1.07	1.59
Total Dispensable amino acids			
Alanine (%)	4.93	1.10	1.74
Aspartic acid (%)	5.16	2.26	2.99
Cysteine (%)	0.55	0.37	0.42
Glutamic acid (%)	7.31	3.85	4.76
Glycine (%)	3.14	0.91	1.32
Proline (%)	2.05	1.25	1.42
Serine (%)	2.03	0.96	1.21
Tyrosine (%)	2.51	1.75	1.16

^1^ Broiler chick grower diet (Table 1) + Titanium dioxide (5 g/kg). ^2^ 75% Broiler chick grower diet (Table 1) + 25% Spirulina + Titanium dioxide (5 g/kg).

**Table 3:** Analyzed energy and nutrient composition (as fed basis) of the spirulina and experimental diets used in the laying hen digestibility trial. Diets were fed as a mash.

Composition (%, As Fed)	Spirulina	Control Diet ^1^	Test Diet ^2^
Dry matter (%)	87.0	89.5	88.7
Gross energy (kcal/kg)	4569	3430	3785
Apparent metabolizable energy (kcal/kg)	3319	2386	2619
Crude protein (%)	59.5	15.3	25.5
Crude fat (%)	2.4	2.7	2.6
Neutral detergent fiber (%)	3.3	13.8	12.2
Acid detergent fiber (%)	1.3	5.4	3.8
Ash (%)	8.2	13.0	10.5
Non-fiber carbohydrate (%, calculated by difference)	13.6	44.7	37.9
Total Indispensable amino acids			
Arginine (%)	3.32	0.88	1.41
Histidine (%)	0.76	0.41	0.48
Isoleucine (%)	3.28	0.62	1.27
Leucine (%)	4.87	1.42	2.21
Lysine (%)	2.53	0.72	1.11
Methionine (%)	1.34	0.45	0.68
Phenylalanine (%)	2.66	0.75	1.18
Threonine (%)	2.66	0.56	1.03
Tryptophan (%)	0.69	0.18	0.26
Valine (%)	3.58	0.74	1.42
Total Dispensable amino acids			
Alanine (%)	4.69	0.88	1.77
Aspartic acid (%)	4.94	1.27	2.11
Cysteine (%)	0.51	0.25	0.34
Glutamic acid (%)	7.07	2.66	3.56
Glycine (%)	3.02	0.64	1.19
Proline (%)	2.00	1.00	1.19
Serine (%)	2.11	0.65	0.91
Tyrosine (%)	2.32	0.51	0.86

^1^ Laying hen diet (Table 1) + Titanium dioxide (5 g/kg). ^2^ 75% Laying hen diet (Table 1) + 25% Spirulina + Titanium dioxide (5 g/kg).

**Table 4:** Comparison of apparent metabolizable energy (AME) of spirulina (as fed basis) fed to broiler chicks and laying hens in this study. Values are presented as means ± SEM.

	AME	
	(kcal/kg, As Fed)	(% of Gross Energy)
Broiler chick	2368 ± 104	52.2 ± 2.3
Laying hens	3294 ± 181	72.1 ± 4.0
*p* value	*p* < 0.001	*p* < 0.001

**Table 5:** Comparison of apparent ileal digestibility of indispensable and dispensable amino acids of Spirulina (%) fed to broiler chicks and laying hens. Values are presented as means ± SEM.

	Broiler Chicks	Laying Hens	*p* Value
Indispensable amino acids			
Arginine (%)	90.4 ± 2.3	87.4 ± 1.1	0.148
Histidine (%)	76.1 ± 1.3	61.9 ± 9.2	0.099
Isoleucine (%)	76.8 ± 0.9	93.4 ± 7.7	0.065
Leucine (%)	73.9 ± 1.3	76.4 ± 7.2	0.390
Lysine (%)	94.5 ± 1.0	70.3 ± 13.7	0.076
Methionine (%)	91.3 ± 0.6	79.8 ± 6.2	0.091
Phenylalanine (%)	81.3 ± 1.4	77.2 ± 8.7	0.353
Threonine (%)	71.4 ± 3.5	86.4 ± 12.1	0.177
Tryptophan (%)	85.9 ± 1.3	86.7 ± 10.5	0.476
Valine (%)	69.8 ± 2.6	91.0 ± 8.3	0.044
Dispensable amino acids			
Alanine (%)	62.0 ± 2.0	103.0 ± 6.0	0.001
Aspartic acid (%)	79.1 ± 0.4	80.7 ± 8.4	0.440
Cysteine (%)	56.1 ± 1.4	64.0 ± 8.2	0.227
Glutamic acid (%)	82.6 ± 0.4	66.7 ± 7.7	0.070
Glycine (%)	66.7 ± 0.8	90.7 ± 8.2	0.028
Proline (%)	80.5 ± 2.3	70.6 ± 7.5	0.161
Serine (%)	78.1 ± 5.6	74.8 ± 9.2	0.398
Tyrosine (%)	98.5 ± 1.9	83.0 ± 9.7	0.119

## Data Availability

The original contributions presented in the study are included in the article and Appendix A, further inquiries can be directed to the corresponding author.

## Appendix A

**Table A1:** Summary of the gross energy and nutrient composition of spirulina (as fed basis) utilized in the present study (Table 2 and Table 3) and in previous poultry nutrient digestibility trials ^1^.

Composition (% As Is Basis)	Mean ± SEM	Range	CV (%)
Dry matter (%)	88.6 ± 0.9	86.2–92.3	2.4
Gross energy (kcal/kg)	4588 ± 121	4286–5144	6.5
Crude protein (%)	58.6 ± 1.9	51.5–65.4	8.1
Crude fat (%)	1.9 ± 0.4	0.8–3.2	54.2
Crude fiber (%)	2.2 ± 0.5	0.78–5.75	108.1
Neutral detergent fiber (%)	5.7 ± 2.4	3.3–10.6	73.6
Acid detergent fiber (%)	1.1 ± 0.2	0.8–1.3	26.1
Ash (%)	8.1 ± 0.4	7.1–9.4	10.7
Non-fiber carbohydrate (%, calculated by difference)			
Indispensable amino acids			
Arginine (%)	3.60 ± 0.16	3.32–3.92	8.6
Histidine (%)	0.82 ± 0.05	0.73–0.93	11.1
Isoleucine (%)	3.18 ± 0.16	2.71–3.45	10.1
Leucine (%)	4.87 ± 0.17	4.40–5.14	6.9
Lysine (%)	2.56 ± 0.14	2.18–2.78	10.8
Methionine (%)	1.28 ± 0.16	0.81–1.49	24.8
Phenylalanine (%)	2.56 ± 0.11	2.23–2.72	8.8
Threonine (%)	2.71 ± 0.09	2.49–2.94	6.9
Tryptophan (%)	0.64 ± 0.03	0.61–0.69	6.8
Valine (%)	3.34 ± 0.25	2.61–3.75	15.1
Dispensable amino acids			
Alanine (%)	4.30 ± 0.33	3.44–4.93	15.4
Aspartic acid (%)	5.18 ± 0.14	4.94–5.57	5.3
Cysteine (%)	0.73 ± 0.19	0.51–1.31	52.6
Glutamic acid (%)	7.48 ± 0.30	7.07–8.36	8.0
Glycine (%)	2.98 ± 0.07	2.85–3.14	4.4
Proline (%)	2.07 ± 0.04	2.00–2.18	3.7
Serine (%)	2.43 ± 0.22	2.03–2.96	18.0
Tyrosine (%)	2.41 ± 0.12	2.13–2.66	9.6

^1^ Yoshida and Hoshii [8]; Alvarenga et al. [10]; Tavernari et al. [12]; Mullenix [13].

**Table A2:** Comparison of apparent metabolizable energy (AME) values of spirulina (as fed basis) fed to broiler chicks and laying hens.

	AME	
	(kcal/kg)	(% GE)
Broiler chicks		
Alvarenga et al. [10]	2560	59.7
Tavernari et al. [12]	2864	65.2
Mullenix [13]	2913	56.6
Present study (Table 2)	2368	52.2
Leghorn male chick		
Yoshida and Hoshii [8]	3290	71.7
Laying hen		
Present study (Table 3)	3294	72.1
Mean ± SEM	2882 ± 154	62.9 ± 3.3
CV (%)	13.0	12.9

**Table A3:** Comparison of apparent ileal digestibility of indispensable and dispensable amino acids of spirulina (%) fed to broiler chicks and laying hens.

	Broilers			Layers		
	Tavernari et al. [12]	Mullenix [13]	Current Study	Current Study	Average	CV (%)
Indispensable						
Arginine (%)	74.7	75.9	90.4	87.4	82.1 ± 0.04	9.7
Histidine (%)	72.0	67.5	76.1	61.9	69.4 ± 0.03	8.8
Isoleucine (%)	74.7	74.2	76.8	93.4	79.8 ± 0.05	11.5
Leucine (%)	72.4	73.1	73.9	76.4	74.0 ± 0.01	2.4
Lysine (%)	76.8	70.4	94.5	70.3	78.0 ± 0.06	14.6
Methionine (%)	77.2	76.9	91.3	79.8	81.3 ± 0.03	8.4
Phenylalanine (%)	76.1	72.6	81.3	77.2	76.8 ± 0.02	4.7
Threonine (%)	72.4	71.3	71.4	86.4	75.4 ± 0.04	9.8
Tryptophan (%)		62.0	85.9	86.7	78.2 ± 0.08	17.9
Valine (%)	72.5	83.2	69.8	91.0	76.3 ± 0.05	13.0
Dispensable						
Alanine (%)	73.6	75.0	62.0	103.0	78.4 ± 0.09	22.2
Aspartic acid (%)	68.7	73.7	79.1	80.7	75.6 ± 0.03	7.2
Cysteine (%)	68.7	60.0	56.1	64.0	62.2 ± 0.03	8.7
Glutamic acid (%)	74.5	74.2	82.6	66.7	78.5 ± 0.02	6.1
Glycine (%)	66.9	71.9	66.7	90.7	74.1 ± 0.06	15.3
Proline (%)	74.2	71.7	80.5	70.6	74.3 ± 0.02	6.0
Serine (%)	68.2	68.5	78.1	74.8	72.4 ± 0.02	6.7
Tyrosine (%)	70.5	59.0	98.5	83.0	77.8 ± 0.08	21.8
